# Effective approaches to public engagement with global health topics

**DOI:** 10.7189/jogh.10.010901

**Published:** 2020-06

**Authors:** Iain H Campbell, Igor Rudan

**Affiliations:** Centre for Global Health, Usher Institute, University of Edinburgh, Edinburgh, Scotland, UK

## Abstract

**Background:**

“Public engagement” in science is a term that covers a broad spectrum of activities undertaken by the scientific community. The precise definitions are constantly evolving to incorporate new means of engagement, facilitated by emerging technologies. Global health research is amenable to community engagement and popularization, but it is difficult to know which strategies work best to attract considerable attention from the public.

**Methods:**

This is a review of the articles and documents that address the question of public engagement with topics in medical sciences, particularly in global health. Semantic searches were conducted using Google Scholar rather than indexed databases due to poor indexing of the topic. More than 1000 titles were screened and 48 articles were retained as most useful. It then moves to a more specific topic of the online public engagement in global health.

**Results:**

The review presents the attempts to define public engagement in science and its general importance, particularly in the field of global health. Examples of the latter include tobacco use, vaccination, and maternal and child health. In reviewing effective approaches to public engagement in global health through online video campaigns, it studies the examples of crowdfunding, USAID’s First Public Engagement Campaign, World Health Organization's Social Media Campaigns and the impact of Global Health Media Project.

**Conclusions:**

This review reveals three key gaps in the understanding of determinants of effective online public engagement in global health. The mixed results of traditional mass media campaigns in global health emphasise the calls for more research on message content. A framework for effective message content would help in both raising awareness of key issues and creating behaviour change in the general public. Moreover, it is surprising to find no formal research on what constitutes effective video content in global health. Finally, few studies considered important metrics to track in social media campaigns. There is a clear need to investigate which video features are effective in global health online public engagement. Success will be defined through key video marketing metrics and tracked in order to isolate effective content features.

## DEFINITION OF PUBLIC ENGAGEMENT IN SCIENCE

“Public engagement” in science is a term that covers a broad spectrum of activities undertaken by the scientific community. The precise definitions are constantly evolving to incorporate new means of engagement, facilitated by emerging technologies. However, several key organisations that promote public engagement have provided some broad definitions.

The National Co-ordinating Centre for Public Engagement (NCCPE) defines public engagement as: “the myriad ways in which the activity and benefits of higher education and research can be shared with the public. Engagement is by definition a two-way process, involving interaction and listening, with the goal of generating mutual benefit” [1],

The Higher Education Funding Council for England further emphasises the “two-way” process of Public Engagement with a more precise definition of the “public”:

“‘Public engagement involves specialists in higher education listening to, developing their understanding of, and interacting with non-specialists. The ‘public’ includes individuals and groups who do not currently have a formal relationship with an HEI through teaching, research or knowledge transfer” [2].

The Research Councils UK public engagement concordat defines public engagement as a diversity of activities including [3]:

- Participating in festivals
- Working with museums / galleries / science centres and other cultural venues
- Creating opportunities for the public to inform the research questions being tackled
- Researchers and public working together to inform policy
- Presenting to the public (e.g. public lectures or talks)
- Involving the public as researchers (e.g. web based experiments)
- Engaging with young people to inspire them about research (eg, workshops in schools)
- Contributing to new media enabled discussion forums.

This definition published in 2010 could be expanded to include novel methods of Public Engagement such as Massive Open Online Courses and other e-learning methods which are rapidly increasing in popularity [4].

## IMPORTANCE OF PUBLIC ENGAGEMENT IN SCIENCE

In recent years many of the institutions that govern and fund public research have called for increased participation in public engagement [5]. This call has been supported by a number of large public engagement grants from organisations such as The Wellcome Trust and the Science and Technologies Facilities Council (STFC).

In 2008 Research Councils UK and The Wellcome Trust established the National Coordinating Centre for Public Engagement with an aim to “support a culture change in the HEI sector” [6]. The increasing importance of translation of research results towards the benefits of the wider general public, including public engagement, was recognised in the 2014 Research Excellence Framework which awards 20% of the overall score to “impact”. Thereby, impact was very broadly defined as ‘an effect on, change or benefit to the economy, society, culture, public policy or services, health, the environment or quality of life, beyond academia’. [7] Research Councils UK now require a 2-page “Pathways to Impact” statement with all bids [8] and have published a Concordat for Engaging the Public With Research [2]. As of 2010 The European Commission has recognised public engagement as the most important factor in its “Responsible Research and Innovation” and “Science with and for Society” frameworks [9].

These developments have led to the institutionalisation of public engagement in many higher education institutes. However, many researchers are still unsure of how to participate effectively. In December 2015, a Consortium of 16 funders of UK public research commissioned a research study to investigate the current landscape of public engagement by researchers in higher education, research institutes and clinical settings. The consortium concluded that while participation and value placed on public engagement has increased in recent years, “researchers and institutions remain uncertain about public engagement, within the context of a profession that is driven by research (and teaching)” [10].

The majority of researchers within science, technology, engineering and mathematics participate in public engagement [11]. However, despite encouragement from large funding bodies such as the Wellcome Trust to increase participation, many academics still do not regard science communication as one of the central aspects of academic life [11]. A 2006 Royal Society Survey of factors affecting science communication based on a sample of researchers in science, technology, engineering, and mathematics reported that 74% of the scientists surveyed had taken part in public engagement activities within the previous 12 months and 70% agreed that funders should encourage such activities. However 64% reported that the need to focus primarily on research prevented them from becoming more engaged [11].

With the rapid evolution of digital technologies and communication, the higher education and research sectors have been relatively slow to utilise social media [12]. This has created confusion around the place of public engagement in these sectors, which have themselves undergone broad structural changes in recent years. As a result of insufficient science communication, the public’s perception of many key scientific issues varies considerably from that of the scientific community [5]. A study by The Pew Research group in association with the American Association for the Advancement of Science (AAAS) illustrated this difference of opinion with regard to biomedical science. For example, the gap between citizen and scientist opinions on whether genetically modified foods are safe to eat differs by 51% [5]. A recent article in Nature concluded: “this strategy of isolation is not effective in today’s world...In the face of a rapidly changing society, science researchers must learn from Darwin and evolve” [13]. Similarly, the Academy of Medical Sciences April 2016 report to the House of Commons on Science Communication concludes:

“For the UK to continue to lead the world in scientific research, we must deliver communications that demonstrate science is at the heart of British culture. This requires a concerted effort on the part of scientists, the media, Government and society as a whole to promote conversations about the scientific method, its benefits and shortcomings” [14].

The Pew Research study highlighted both a desire on the part of scientists to engage with the public and dissatisfaction with current media coverage of scientific findings. 79% of the scientists surveyed believe that inaccurate media reporting is a major problem for science [5].

While scientists increasingly view interaction with the public through the media and social media as an important means to advance their career, the majority still consider it to be a secondary activity to other academic commitments [5].

## IMPORTANCE OF PUBLIC ENGAGEMENT IN GLOBAL HEALTH

Cohen et al [15] list several key benefits that public engagement can provide to the field of global health. These include creating an informed citizenry, generating new ideas from the public, increasing the chances of research being adopted, increasing public trust, answering ethical research questions, fostering global communication, enabling shared experiences and methodology, standardization of strategy and generating global viewpoints.

Increasing sensitivity to global health topics among the high-income countries holds the promise of increasing focus on many neglected diseases and themes, and even raising funding towards global health interventions through crowd-funding campaigns.

Until the 1990s, public engagement in global health was primarily carried out through in-person events and the mass media (radio and television). As technology quickly evolves many of these campaigns are moving to online platforms. There are many lessons to be learned from mass media campaigns in global health, which are applicable to newer online campaigns. This review will examine 3 important areas where Mass Media campaigns have been implemented in global health interventions: Tobacco Use, Vaccination Adoption, and Maternal and Child Health.

### Tobacco use and public engagement

From the 1970s to the mid 1990s many mass media campaigns were carried out to educate the public about the risks of tobacco use. A report by the National Cancer Institute in 2008 attributes a significant contribution of media campaigns to the reduction of per capita cigarette consumption by approximately one half since the peak in the 1960s [16]. Systematic reviews support this assertion [17,18]. However Wakefield et al. [17] identify the lack of formal control groups as an important limitation of evaluating mass media interventions. This limitation makes it difficult to isolate which campaigns produced an effect. However, the overall benefit is clearly demonstrated when such campaigns are discontinued and the beneficial effects are seen to decrease [19]. Clearly, online campaigns that can be highly targeted will be able to overcome such limitations.

While a strong benefit of engagement with the public through mass media has been observed, less is understood about what kinds of messages are most effective. Message content is highly important in such interventions as demonstrated by the “anti-smoking” campaigns of Phillip Morris, which actually increased the likelihood of young people smoking in the future [20]. The role of grounding in social science when designing message content has been strongly emphasised by social scientists [21]. The leading role of media campaigns in the fight against tobacco use led the World Health Organization (WHO) to conclude that “Tobacco addiction is a communicated disease - communicated through advertising, sports, marketing and sponsorship” [22]. Clearly, message design should be taken as seriously in global health campaigns as it is in commercial marketing. Tobacco company counter campaigns repeatedly used their expertise in this area to their advantage throughout the 1980s [18].

### Vaccination and public engagement

Media coverage of influenza vaccinations is associated with increased vaccination rates and earlier adoption by the public [23]. The importance of scientists engaging with the public on such issues has been emphasized by a qualitative investigation of vaccine risk perception, which reports that “parents lack trust in government agencies and may have doubts in the medical profession as the ‘managers’ of vaccine risk” [24].

Each year the WHO promotes a “World Vaccination Week” as a core part of its Global Vaccine Action Plan (GVAP) [25]. Videos, graphics and other social media content are distributed during the week to raise awareness. The GVAP is currently not on track to achieve its ambitious goals. However, the SAGE 2015 assessment reports “A WHO review of the countries that have successfully eliminated maternal and neonatal tetanus put a high value on early and active community engagement” [26].

As early as 1987 Heggenhougen et al. asserted that the social science surrounding vaccine adoption “has been a closed book to immunization program managers for too long” [27]. More recently, prominent media experts have emphasized the importance of message content in achieving vaccination adoption in the public [28]. In recognition, the WHO has published detailed guidelines on message content for media promotion of vaccination [29]. Despite recognition in the Global Vaccine Action Plan that “Communications and social research to identify the barriers to and drivers of vaccination should inform the development of context-specific messages” there is no evidence of such research underpinning the WHO guidelines [25].

The influence of the media on vaccine adoption in the public is demonstrably profound. Despite increasing access to vaccination, the GVAP has thus far struggled to translate this power into the levels of adoption necessary to meet their goals. If lessons are to be learned from the success of tobacco interventions then interaction with social science and consideration of message design will continue to be important factors for success.

### Maternal and child health and public engagement

Many traditional mass media campaigns have been employed in an effort to improve maternal and child health, but the results have been mixed.

A 2014 systematic review of mass media campaigns and child survival reported evidence of behaviour change in 26 of the 32 studies [30]. The campaigns promoted a wide diversity of behaviours, including antenatal care, vaccine uptake and use of oral rehydration sachets. Naugule et al. report that media intervention alone is not enough to secure behavioural change, but that as a supplement to community based initiatives they increase the uptake significantly [31].

In one of the most rigorously designed mass media interventions using radio in Burkina Faso over 20 months, little evidence for significant behaviour change was observed despite the heavy broadcasting of the health messages [32]. It is suggested in the study that this may be due to strongly entrenched community beliefs and practices, which are hard to shift through media exposure alone.

From the overwhelmingly positive results of mass media anti-smoking campaigns and the measurable success of commercial advertising campaigns in the western world, it is apparent that such campaigns can change population opinions and behaviours. However, many trials of such strategies in other areas of global health have found mixed results. Perhaps the key difference is the role and reach of the media in the high-income vs low- and middle-income countries. Most of the anti-tobacco campaigns were carried out in America, whereas vaccination and maternal and child health campaigns are primarily carried out in low and middle income countries where media tends to hold less influence.

## ONLINE PUBLIC ENGAGEMENT IN GLOBAL HEALTH

A picture emerges that significant media reach must be combined with sensitive messaging rooted in social science, as well as follow up and interaction with the public in which behaviour change is to be affected. These insights reveal a key limitation of mass media public engagement campaigns. Television and Radio are for the most part a one-way form of communication. Considering that the NCCPE emphasise the two-way nature of effective Public Engagement, these platforms are beginning to look outdated. Online platforms allow for immediate feedback and engagement with the public through interactive elements such as surveys, e-mail correspondence and social media. Social Science research emphasises the importance of relationships among community members as a key factor in behavioural change [33]. Online campaigns can instigate conversations and action among communities in a way that one-way radio and television communication cannot.

Additionally, the barriers to access and cost associated with online campaigns are considerably lower. Digital analytics allow for precise tracking of campaign goals and a deeper understanding of which features of a campaign are working. Online digital advertising allows a researcher to target a highly specific audience with media information, bypassing nearly all geographical or socio-economic restrictions associated with traditional mass media approaches. The cost of engaging the public in this manner is very low and budgets can be utilised with great efficiency. For example a video summarising the findings of a research project relevant to Asthma sufferers could be highly targeted to an audience through Google Ad-words. This audience can be defined by hundreds of factors including age, sex, location and search preferences (“Asthma advice” “Inhalers” “Difficulty breathing” etc.) for less than 30 pence per view. In this way every penny spent gets the information in front of the relevant audience in a direct manner.

These advantages address the three main concerns of the 2015 Public Engagement Consortium report [10]. They make public engagement impact measurable, the automation saves time for busy researchers who currently view public engagement only as a duty and the digital tools make public engagement more simple for researchers who are still adapting to the current online media landscape.

In recognition of this changing landscape, The Academy of Medical Sciences 2016 report to the House of Commons advises: “science communication should target the broadest possible audience, requiring innovative strategies to engage hard-to-reach groups” [14].

## EFFECTIVE APPROACHES TO PUBLIC ENGAGEMENT IN GLOBAL HEALTH THROUGH ONLINE VIDEO CAMPAIGNS

### Video and crowdfunding

Global fiscal austerity measures and increasing competition are driving some global health scientists to seek crowdfunding as an alternative means of financing research [34]. Crowdfunding holds the promise of fuelling greater innovation in global health research by providing alternative funding for innovative projects that would not appeal to traditional funding bodies. Freedom from the time constraints of grant cycles could allow for faster innovation and evolution of technology. Established funding models are well suited to high impact large-scale health campaigns, but do not often encourage the kind of disruptive innovation the technology start-up world facilitates. The average age at the first grant award from the National Institutes of Health is 42 for PhDs [35]. In contrast, the average age of a start-up founder in Silicon Valley is 31 [36]. Crowdfunding has the potential to democratise scientific research, but there is also danger in its funding only so-called “panda-projects”, which appeal emotionally to the crowd but have less scientific merit.

The majority of current global health crowdfunding is based around sponsoring doctors and treatments in low and middle income countries through existing non-profit structures. This method is often referred to as crowd-sourcing. The primary appeal of this model is the transparency that allows a donor to see exactly where every penny of their donation is spent as they are put in touch with the doctor they sponsor. The founders of these projects agree that the main appeal of their model is transparency [37,38]. The donors are sent updates directly from the doctor providing treatment on the progress of the patient. In all of these models 100% of the money donated is spent directly on patient treatment.

Watsi [37] and Crowd Fund Health [38] are 2 examples of crowdfunding platforms which identify low-cost, high-impact treatments, crowdfund the cost and partner with a medical service provider to deliver the treatment.

Kickstarter - the world’s leading crowdfunding platform - is driven primarily through the viral sharing of campaign videos. Campaigns using a video are 66% more likely to be funded than those without [39]. Most global health crowdfunding platforms use video to support their campaigns. *However, there is currently no research on what constitutes an effective video.* There are many blog articles that attempt to break down which features contribute to success of crowdfunding videos, but few are backed by the kind of social science research that has been a characteristic of previously successful media campaigns in global health.

### USAID’s first public engagement campaign

Many large international agencies, bilateral agencies, non-governmental organisations and higher education institutions have also embraced use of online video in their campaigns over the last decade. In 2011 USAID launched the FWD online campaign in an effort to raise public awareness of famine in the horn of Africa [40]. The campaign focused on three main objectives:

“Deliver results on a meaningful scale through a strengthened USAID…Promote sustainable development through high-impact partnerships and local solutions…Identify and scale up innovative, breakthrough solutions to intractable development challenges” [41].

The campaign created direct engagement with the U.S public through a television campaign, social media campaign and special events. The project is regarded as a highly effective intervention raising over $5 million in private donations and leveraging billions more worldwide through new and existing partnerships [41]. The campaign had a significant social media impact, generating 150 million “forwards”, page-views, “likes” and friends through Facebook, re/tweets on Twitter, and YouTube views [41].

Despite the overwhelming success of USAIDs first major public engagement endeavour, many unique online strategies were employed which do not have standardised assessment protocols and as a result the true impact of the campaign is difficult to measure [41]. In reference to the FWD campaign, Abdullateef comments “While these campaigns frequently enjoy success, the effectiveness of measuring “public engagement” has been an ongoing challenge” [41]. Abdullateef views grounding in social science as a key factor of the success of the project. The campaign sought to identify leaders in the targeted social groups and reach them first as opposed to the “scatter gun” approach of many other campaigns.

If similar positive results are to be obtained by further global health public engagement campaigns, then key metrics in online public engagement must be established to facilitate goal-setting and analysis. Strategies incorporating a deep understanding of social science were identified to be a key feature of campaign success in this example.

### WHO social media campaigns

The WHO has launched several social media campaigns over the past 3 years. In 2016 it launched “Get Healthy” in order to “engage the Chinese public in a conversation about how a healthy lifestyle can help guard against disease” [42].

The study, hosted on micro-blogging platform Weibo shares basic healthy eating advice through pictures and videos. A similar campaign on Weibo was launched in 2015 called “Got it Covered” with an aim to share safe sex information with college and university students [43].

The WHO has incorporated social media activities into its Global Vaccine Action Plan. World immunisation week online engagement activities include an online quiz, infographics, video, twitter chat and Facebook Q&A. However the objectives in the Global Vaccine Action Plan are very vague, stating only that: “New efforts could take advantage of social media and approaches used by commercial and social marketing efforts to promote immunization and address concerns” [29].

### Global Health Media Project

The Global Health Media project has made a significant contribution to the use of online video in global health [44]. Their videos have received a total of 13 millions views world-wide as of September 2016 clearing demonstrating the need for well produced global health video content [45].

Their work falls into 2 main categories: 1. Training videos for health care workers and 2. Short films which raise awareness of key global health topics. Their most popular short film “The Story of Ebola” produced in collaboration with the International Federation of Red Cross and Red Crescent Societies and UNICEF, is a short animated video over 1 million views [46]. And their most popular training video is a video on increasing milk supply in Swahili, with over 6 million views [47].

Their work highlights 2 clear needs in global health which can be served by video: Awareness raising films and instructional videos for health care workers.

Their videos are used by primarily by NGOS (40% of video downloads), teaching institutes (23%) and health facilities (14%) [45].

Despite the success of these videos there is no published research on why such videos have become so successful compared with other global health video content. An analysis of the success of these videos could contribute to setting best-practice guidelines for global health organisations who wish to engage the public on similar topics [48].

## GAPS IN RESEARCH ON EFFECTIVENESS OF PUBLIC ENGAGEMENT IN GLOBAL HEALTH

This review of the literature reveals three key gaps in the understanding of determinants of effective online public engagement in global health.

1. The mixed results of traditional mass media campaigns in global health emphasise the calls for more research on message content from social scientists. A framework for effective message content would benefit many organisations in global health in both raising awareness of key issues and creating behaviour change in the public.

2. Online video is the most important communication tool of many modern global health public engagement projects holding a primacy in both crowdfunding and social media campaigns. Therefore, it is surprising to find no formal research on what constitutes effective video content in global health.

3. Only one study in this literature review considered important metrics to track in social media campaigns [40]. Compared with the sophistication with which commercial marketing campaigns are carried out with hundreds of key metrics tracked; global health campaigns seem to be “shooting in the dark.”

There is a clear need to investigate which video features are effective in global health online public engagement. Success will be defined through key video marketing metrics and tracked in order to isolate effective content features. These studies will be grounded in, and informed by quantitative social science research.
